# The Importance of Brain Metastasis in EGFR Mutation Positive NSCLC Patients

**DOI:** 10.1155/2014/856156

**Published:** 2014-12-07

**Authors:** Vanita Noronha, Amit Joshi, Anant Gokarn, Vibhor Sharma, Vijay Patil, Amit Janu, Nilendu Purandare, Anuradha Chougule, Nirmala Jambhekar, Kumar Prabhash

**Affiliations:** ^1^Department of Medical Oncology, Tata Memorial Hospital, Dr. E. Borges Road, Parel, Mumbai, Maharashtra 400012, India; ^2^Department of Medical Oncology, Malabar Cancer Center, Moozhikkara, Kannur, Kerala 670103, India; ^3^Department of Radiodiagnosis, Tata Memorial Hospital, Dr. E. Borges Road, Parel, Mumbai, Maharashtra 400012, India; ^4^Department of Nuclear Medicine, Tata Memorial Hospital, Dr. E. Borges Road, Parel, Mumbai, Maharashtra 400012, India; ^5^Department of Pathology, Tata Memorial Hospital, Dr. E. Borges Road, Parel, Mumbai, Maharashtra 400012, India

## Abstract

*Introduction*. Brain metastasis is a poor prognostic marker in lung cancer. However it is not known whether amongst patients with EGFR mutation those with brain metastases have a worse outcome. *Methods*. We compared the survival outcomes between EGFR mutation positive patients with and without brain metastases. In this retrospective analysis of prospective database of all metastatic lung cancer patients at our centre between July 2009 and December 2012, patients were treated with either combination chemotherapy or oral TKI. All patients with brain metastases received whole brain radiation. Kaplan Meier method was used for survival analysis and compared using log rank test. *Results*. 101 patients with EGFR mutated, metastatic lung cancer were studied. Fourteen had brain metastases and 87 did not. The common EGFR mutations were exon 19 deletion (61.3%) and exon 21 L858R mutation (28.7%). Overall response was 64% in extracranial metastasis group as compared to 50% in brain metastasis group. There was a significant worsening of median OS in the patients with brain metastases (11.6 months) compared with only extracranial metastases (18.7 months), *P* = 0.029. *Conclusion*. Amongst patients with EGFR mutant NSCLC, the presence of brain metastases leads to a worse outcome as compared to patients with extracranial metastases alone.

## 1. Introduction

Metastatic lung cancer is one of the leading causes of cancer mortality worldwide. The presence of brain metastasis confers an even worse prognosis [1]. The median survival amongst patients with adenocarcinoma of the lung with brain metastasis in one of the early reports was around 73 days [1]. Whole brain radiotherapy (WBRT) improves median survival to 4–6 months [2, 3]. Non-small cell lung cancer (NSCLC) patients with brain metastases who have activating mutations of epidermal growth factor receptor (EGFR) tend to do significantly better as compared to those with wild type EGFR (median survival of 12.9 months as compared to 3.1 months) [4], when treated with oral tyrosine kinase inhibitors (TKIs) and cranial irradiation.

EGFR mutation positivity is a good prognostic marker and patients with EGFR mutant lung cancer tend to have a longer survival. However, patients with EGFR mutated NSCLC have a predilection to develop brain metastases. The incidence of EGFR mutation positivity among patients with brain metastases is higher, ranging from 44 to 63%, as compared to the usually described 10% incidence of EGFR mutation in all patients diagnosed with NSCLC [5]. Although the development of brain metastases in general predicts for a poor outcome in lung cancer, it is not known whether among patients who are EGFR mutation positive the subset of patients who develop brain metastases have an equally poor prognosis as compared to those EGFR mutation positive patients who have extracranial metastasis only. Hence, we performed a retrospective analysis to try to evaluate whether the presence of brain metastasis amongst patients with EGFR mutations is associated with a worse outcome as compared to those without brain metastasis.

## 2. Materials and Methods

This study was approved by the Institutional Ethics Committee of Tata Memorial Hospital.

### 2.1. Patients

Patients were selected retrospectively from the database maintained prospectively in the Medical Oncology Department of Tata Memorial Hospital, Mumbai (India). All EGFR mutation positive patients with metastatic NSCLC, between July 2009 and December 2012, were included in this study. EGFR mutation test was done as published previously [6].

The diagnosis of NSCLC was made in all patients by means of either a lung biopsy or pleural fluid cytology. Staging workup included a CT scan or PET/CT scan as per routine care. Brain imaging was done only if patient had symptoms or signs suggestive of brain metastasis at the time of diagnosis.

Therapy was administered according to the treating physician's discretion. All patients who were willing to wait for the results of the EGFR analysis report and were not severely symptomatic were started on treatment after the results of the EGFR analysis report were available. The oral tyrosine kinase inhibitor (TKI) used was either gefitinib 250 mg once daily or erlotinib 150 mg once daily, based on the treating physician's preference. Patients with a good performance status (PS), that is, ECOG 1 or 2 but who were very symptomatic because of disease-related symptoms, were started immediately on 1st line chemotherapy with pemetrexed and platinum, prior to obtaining the results EGFR report. Patients who had a poor PS (ECOG 3 or 4) were initially started on oral TKIs, without waiting for the results of EGFR analysis. All patients with brain metastasis received WBRT. Patients were evaluated 1 week after starting oral TKI and then once every 1–3 months to evaluate for side effects. Response evaluation was done every 2-3 months. Response was assessed by using RECIST criteria. Patients who were diagnosed with symptomatic brain metastases were treated with WBRT followed by either chemotherapy (pemetrexed-platinum) or oral TKI, based on their performance status and/or EGFR mutation status. For the purpose of this study, the outcome of patients with brain metastases at diagnosis was compared to that of patients without brain metastases.

### 2.2. Variables

The following demographic and clinical details were captured from the prospective patient database-age, gender, baseline performance status, addictions, histopathology, sites of metastases, type of EGFR mutation, first line therapy, and toxicities to TKIs. Treatment details were collected for these patients. Patient outcome data were collected for response to therapy, progression free survival, and overall survival. All toxicities were graded according to the common terminology criteria for adverse events (CTCAE), version 4.0. Patients who had complications such as febrile neutropenia and skin reactions were managed according to standard protocols as per the discretion of the treating physician.

### 2.3. Statistics

Statistical Package for the Social Sciences (SPSS), version 15.0, was used for compilation of the data as well as for statistical calculation. Frequencies and descriptive statistics were obtained. The categorical variables were compared using chi square method and the Fisher exact test. Overall survival (OS) was defined as the time from date of start of TKI to death. Progression free survival (PFS) was defined as the interval between the date of start of TKI to progression or date of stopping of treatment due to any cause. The OS and PFS were estimated using the Kaplan Meier method and were compared using the log rank test.

## 3. Results

101 EGFR positive patients were included in this study. 14 had brain metastasis. The baseline demographic data are provided in Table 1.

The male to female ratio was equal in patients with brain metastasis but was 0.81 in patients with extracranial metastasis. Extracranial sites of metastases included pleural effusion in 36 patients, bones in 21 patients, and liver in 4 patients. Addictions, both tobacco chewing and smoking, were more common in patients without brain metastases as compared to those with brain metastases (36% versus 28%), although this was not statistically significant.

All patients were exposed to TKI, 70 patients in the first line, 30 in the second line, and one patient received oral TKI as third line therapy. Amongst the patients with brain metastases, TKI was used as 1st line systemic therapy in 6 patients (42.85%), and as 2nd line in 7 patients (50%), while amongst those with extracranial mets, TKI was used as first line in 64 patients (73.5%) and as 2nd line in 23 patients (26.43%).

The most common EGFR mutation seen was exon 19 deletion, which was seen in 62 patients (61.38%), followed by exon 21 L858R mutation in 29 patients (28.71%), and exon 18 G719X in 4 patients. For 3 patients, the EGFR mutation locus was not known as the EGFR mutation testing was done outside our institution: 1 patient had exon 19 mutation and 2 patients had exon 20 mutations.

### 3.1. Outcome

**Figure 1:**
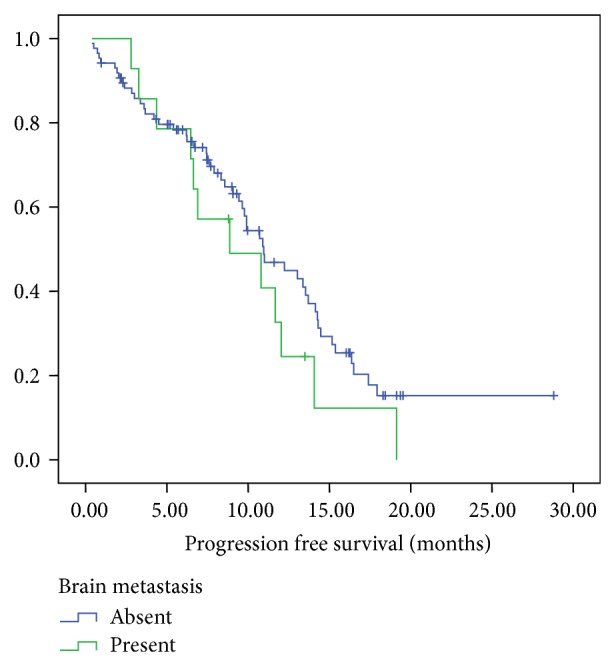
Progression free survival of EGFR positive patients with brain metastases as compared to EGFR positive patients without brain metastases.

**Figure 2:**
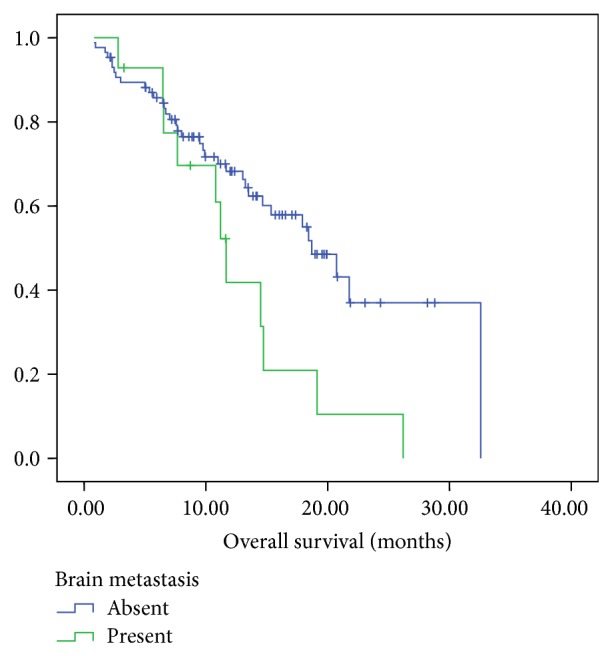
Overall survivals of EGFR positive patients with brain metastases as compared to EGFR positive patients without brain metastases.

In the patients with brain metastases, the overall response rate to first line therapy in the metastatic setting was 50% (all partial responses). In the patients without brain metastases (*n* = 87), the overall response (complete and partial response) rate was 64%. The median PFS was 10.9 months for the patients without brain metastasis as compared to 8.67 months for those with brain metastasis (*P* = 0.237) (Figure 1). However, there was a statistically significant difference between median OS amongst the patients without brain metastases (18.7 months) compared to 11.6 months in the patients with brain metastases, *P* = 0.029 (Figure 2).

## 4. Discussion

Metastatic lung cancer patients with EGFR mutations have a better prognosis as compared to EGFR mutation negative patients. The median survival of EGFR positive patients is around 3 years as compared to 1.6 years in EGFR negative patients, when adjusted for age, gender, and stage. The median survival of lung cancer patients with brain metastasis is 4.5 months when treated with standard WBRT. Patients with brain metastases and EGFR mutations have a higher response rate to WBRT compared to those with wild-type EGFR [7, 8]. However most of the trials evaluating the prognostic role of EGFR have either excluded newly diagnosed symptomatic brain metastasis from their study [9, 10] or included only those with stable and completely treated brain metastases, that is, those who had completed brain irradiation more than 3 weeks prior to enrollment and those who were off corticosteroids [11]. In our study, amongst the EGFR positive patients, survival for those with brain metastasis was significantly worse than those with extracranial metastases alone.

Chemotherapy was believed to have a limited role in patients with brain metastases, due to the general perception that chemotherapy does not cross the blood brain barrier. However, trials have shown that chemotherapy improves median survival in patients with brain metastases [12]. TKIs have also been shown to be effective in the treatment of brain metastases with improvement in OS and PFS [13, 14]. TKIs have also been used upfront without WBRT in patients who are asymptomatic for brain metastases [15–18]. In one of the largest retrospective series examining the effect of EGFR mutations on brain metastasis, it was found that extra cranial progression rather than progression of intracranial metastases was more common in EGFR mutant tumors and these patients were more likely to die from systemic causes rather than brain metastasis when treated with TKIs [13]. Thus the behavior of brain metastatic disease in EGFR mutant patients is different from that of wild type tumors. In our study we found that the overall survival in patients with EGFR mutant NSCLC with extracranial metastases was significantly better than that in patients with brain metastases (18.7 months versus 11.6 months). The overall survival of patients with EGFR mutant NSCLC with brain metastases was similar to that reported in other studies [19]. Although brain metastasis confers a poorer prognosis in lung cancer, this has never been documented in EGFR mutant tumors in the TKI era. In our study, we show that even in EGFR mutant patients treated with TKIs the presence of brain metastasis still leads to worse outcomes as compared to those without brain metastasis.

## 5. Conclusion

Amongst patients with metastatic EGFR mutation positive lung cancer, the patients with brain metastases have a worse outcome as compared to those without brain metastasis at the time of diagnosis.

## Figures and Tables

**Table 1:** Baseline demographic data.

	With brain metastasis, *n* = 14 (%)	Without brain metastasis, *n* = 87 (%)	Total, *n* = 101 (%)	*P* value
Median age (±SD years)	54 (±19.09)	59 (±10.2)	60 (±10.39)	
Gender				
Male	7 (50)	39 (44.82)	46 (45.54)	*P* = 0.13 (chi square)
Female	7 (50)	48 (55.17)	55 (54.45)	
Performance status				
ECOG PS 0-1	5 (35.714)	55 (63.21)	60 (59.4)	*P* = 0.05 (chi square)
ECOG PS 2-3	9 (64.28)	32 (34.48)	41 (40.6)	
Smoking				
Ever	4 (28.57)	32(36.78)	36 (35.64)	*P* = 0.39 (Fisher exact)
Never	10 (71.42)	55 (63.21)	65 (64.35)	
Histology				
Adenocarcinoma	14 (100%)	82 (94.25)	96 (95.04)	*P* = 0.34 (Fisher exact)
Squamous	0	2 (2.29)	2 (1.98)	
Not determined	0	3 (3.44)	3 (2.97)	

## References

[1] Sorensen J. B., Hansen H. H., Hansen M., Dombernowsky P. (1988). Brain metastases in adenocarcinoma of the lung: frequency, risk groups, and prognosis. *Journal of Clinical Oncology*.

[2] Chidel M. A., Suh J. H., Greskovich J. F., Kupelian P. A., Barnett G. H. (1999). Treatment outcome for patients with primary nonsmall-cell lung cancer and synchronous brain metastasis. *Radiation Oncology Investigations*.

[3] Kaal E. C. A., Niël C. G. J. H., Vecht C. J. (2005). Therapeutic management of brain metastasis. *The Lancet Neurology*.

[4] Porta R., Sánchez-Torres J. M., Paz-Ares L. (2011). Brain metastases from lung cancer responding to erlotinib: the importance of EGFR mutation. *European Respiratory Journal*.

[5] Bhatt V. R., Kedia S., Kessinger A., Ganti A. K. (2013). Brain metastasis in patients with non-small-cell lung cancer and epidermal growth factor receptor mutations. *Journal of Clinical Oncology*.

[6] Chougule A., Prabhash K., Noronha V., Joshi A., Thavamani A., Chandrani P., Upadhyay P., Utture S., Desai S., Jambhekar N., Dutt A. (2013). Frequency of *EGFR* mutations in 907 lung adenocarcioma patients of Indian ethnicity. *PLoS ONE*.

[7] Gow C. H., Chien C. R., Chang Y. L., Chiu Y. H., Kuo S. H., Shih J. Y., Chang Y. C., Yu C. J., Yang C. H., Yang P. C. (2008). Radiotherapy in lung adenocarcinoma with brain metastases: effects of activating epidermal growth factor receptor mutations on clinical response. *Clinical Cancer Research*.

[8] Cappuzzo F., Ardizzoni A., Soto-Parra H., Gridelli C., Maione P., Tiseo M., Calandri C., Bartolini S., Santoro A., Crinò L. (2003). Epidermal growth factor receptor targeted therapy by ZD 1839 (Iressa) in patients with brain metastases from non-small cell lung cancer (NSCLC). *Lung Cancer*.

[9] Shepherd F. A., Pereira J. R., Ciuleanu T., Eng H. T., Hirsh V., Thongprasert S., Campos D., Maoleekoonpiroj S., Smylie M., Martins R., Van Kooten M., Dediu M., Findlay B., Tu D., Johnston D., Bezjak A., Clark G., Santabárbara P., Seymour L. (2005). Erlotinib in previously treated non-small-cell lung cancer. *The New England Journal of Medicine*.

[10] Lee S. M., Khan I., Upadhyay S. (2012). First-line erlotinib in patients with advanced non-small-cell lung cancer unsuitable for chemotherapy (TOPICAL): a double-blind, placebo-controlled, phase 3 trial. *The Lancet Oncology*.

[11] Jänne P. A., Wang X., Socinski M. A., Crawford J., Stinchcombe T. E., Gu L., Capelletti M., Edelman M. J., Villalona-Calero M. A., Kratzke R., Vokes E. E., Miller V. A. (2012). Randomized phase II trial of erlotinib alone or with carboplatin and paclitaxel in patients who were never or light former smokers with advanced lung adenocarcinoma: CALGB 30406 trial. *Journal of Clinical Oncology*.

[12] Kim D.-Y., Lee K.-W., Yun T., Kim D.-W., Kim T.-Y., Heo D. S., Bang Y.-J., Kim N. K. (2005). Efficacy of platinum-based chemotherapy after cranial radiation in patients with brain metastasis from non-small cell lung cancer. *Oncology Reports*.

[13] Eichler A. F., Kahle K. T., Wang D. L., Joshi V. A., Willers H., Engelman J. A., Lynch T. J., Sequist L. V. (2010). EGFR mutation status and survival after diagnosis of brain metastasis in nonsmall cell lung cancer. *Neuro-Oncology*.

[14] Hotta K., Kiura K., Ueoka H., Tabata M., Fujiwara K., Kozuki T., Okada T., Hisamoto A., Tanimoto M. (2004). Effect of gefitinib (“Iressa”, ZD1839) on brain metastases in patients with advanced non-small-cell lung cancer. *Lung Cancer*.

[15] Kim J.-E., Lee D. H., Choi Y. (2009). Epidermal growth factor receptor tyrosine kinase inhibitors as a first-line therapy for never-smokers with adenocarcinoma of the lung having asymptomatic synchronous brain metastasis. *Lung Cancer*.

[16] Asahina H., Yamazaki K., Kinoshita I., Sukoh N., Harada M., Yokouchi H., Ishida T., Ogura S., Kojima T., Okamoto Y., Fujita Y., Dosaka-Akita H., Isobe H., Nishimura M. (2006). A phase II trial of gefitinib as first-line therapy for advanced non-small cell lung cancer with epidermal growth factor receptor mutations. *British Journal of Cancer*.

[17] Jung Y. H., Han C. W., Jung Y. D., Cho Y. Y., Han D. J. (2014). Complete remission of brain metastases in non-small cell lung cancer patients harboring an EGFR mutation treated with tyrosine kinase inhibitor without radiotherapy: a report of 3 cases. *Case Reports in Oncology*.

[18] Jamal-Hanjani M., Spicer J. (2012). Epidermal growth factor receptor tyrosine kinase inhibitors in the treatment of epidermal growth factor receptor-mutant non-small cell lung cancer metastatic to the brain. *Clinical Cancer Research*.

[19] Yi H. G., Kim H. J., Kim Y. J., Han S.-W., Oh D.-Y., Lee S.-H., Kim D.-W., Im S.-A., Kim T.-Y., Kim C. S., Heo D. S., Bang Y.-J. (2009). Epidermal growth factor receptor (EGFR) tyrosine kinase inhibitors (TKIs) are effective for leptomeningeal metastasis from non-small cell lung cancer patients with sensitive EGFR mutation or other predictive factors of good response for EGFR TKI. *Lung Cancer*.

